# Laser interstitial thermal therapy is effective and safe for the treatment of brain tumors in NF1 patients after cerebral revascularization for moyamoya angiopathy: a report on two cases

**DOI:** 10.3389/fneur.2023.1291207

**Published:** 2023-12-08

**Authors:** Lelio Guida, Kevin Beccaria, Sandro Benichi, Manoelle Kossorotof, Olivier Naggara, Marie Bourgeois, Franck Bourdeaut, Samuel Abbou, Volodia Dangouloff-Ros, Nathalie Boddaert, Thomas Blauwblomme

**Affiliations:** ^1^Department of Pediatric Neurosurgery, APHP, Hôpital Necker Enfants Malades, Paris, France; ^2^Université de Paris Cité, Paris, France; ^3^Department of Pediatric Neurology, Hôpital Necker Enfants Malades, Paris, France; ^4^Department of Radiology, GHU Sainte-Anne, Paris, France; ^5^Department of Pediatric Oncology, Institut Curie, Paris, France; ^6^Department of Pediatric Oncology, Institut Gustave Roussy, Villejuif, France; ^7^Department of Pediatric Radiology, APHP, Hôpital Necker Enfants Malades, Paris, France; ^8^Université Paris Cité, UMR 1163, Institut Imagine, Paris, France

**Keywords:** laser interstitial thermal therapy (LITT), pediatrics, oncology, moyamoya, revascularization, case report

## Abstract

**Background:**

The co-occurrence of moyamoya vasculopathy and extra-optic pathway tumors is rare in neurofibromatosis type 1 (NF1), with only four cases described in the literature. Brain surgery in these patients may be challenging because of the risk of brain infarction after skin and dural incision. Given its percutaneous and minimally invasive nature, laser interstitial thermal therapy (LITT) is an ideal option for the treatment of brain tumors in these patients. Here, we report on two patients with NF1 and moyamoya syndrome (MMS) treated for a brain glioma with LITT, after cerebral revascularization.

**Cases:**

The first patient, with familial NF1, underwent bilateral indirect revascularization with multiple burr holes (MBH) for symptomatic MMS. Two years later, she was diagnosed with a left temporal tumor, with evidence of radiologic progression over 10 months. The second patient, also with familial NF1, developed unilateral MMS when he was 6 years old and was treated with MBH. At the age of 15 years, MRI showed a right cingular lesion, growing on serial MRIs. Both patients underwent LITT with no perioperative complications; they are progression free at 10 and 12 months, respectively, and the tumors have decreased in volume.

**Discussion:**

While the association of extra-optic neoplasm and moyamoya angiopathy is seldom reported in NF1, tumor treatment is challenging in terms of both avoiding stroke and achieving oncological control. Here, we show in 2 cases, that LITT could be a safe and effective option in these rare conditions.

## Introduction

MR-guided laser interstitial thermal therapy (MRgLITT) is one of the most recent advances introduced in neurosurgery. Since its approval by the FDA in 2007, it has been successfully used in the fields of neuro-oncology (1), epilepsy (2), and recently functional neurosurgery (3). The most attractive advantage of this technique is the possibility of performing laser ablation of a given cerebral volume through a stereotactic percutaneous trajectory under real-time monitoring of brain temperature. For this reason, LITT has established itself as one of the preferred techniques for deep-seated lesions, in order to avoid brain retraction injuries during open surgery. Furthermore, its percutaneous and minimally invasive nature makes it a suitable option for superficial cortical lesions in patients with contraindications to traditional craniotomy (4). In pediatric neuro-oncology, LITT has been shown to be effective for the treatment of brain tumors in a recent multicenter study on 86 children, which reported a progression-free survival rate of 92% at 72 months (5).

Herein, we report the use of LITT in two cases of pediatric patients with neurofibromatosis type 1 (NF1) who presented with the very rare association of moyamoya syndrome (MMS) and extra-optic pathway gliomas [only 4 cases of which are described in the literature (6–9)]. The rationale behind the use of LITT was to minimize the risk of perioperative stroke as a result of the interruption of spontaneous collaterals and external–internal carotid artery anastomoses, which may occur during open brain surgery at the skin or dural incision.

## Case description

### Case 1

**Figure 1:**
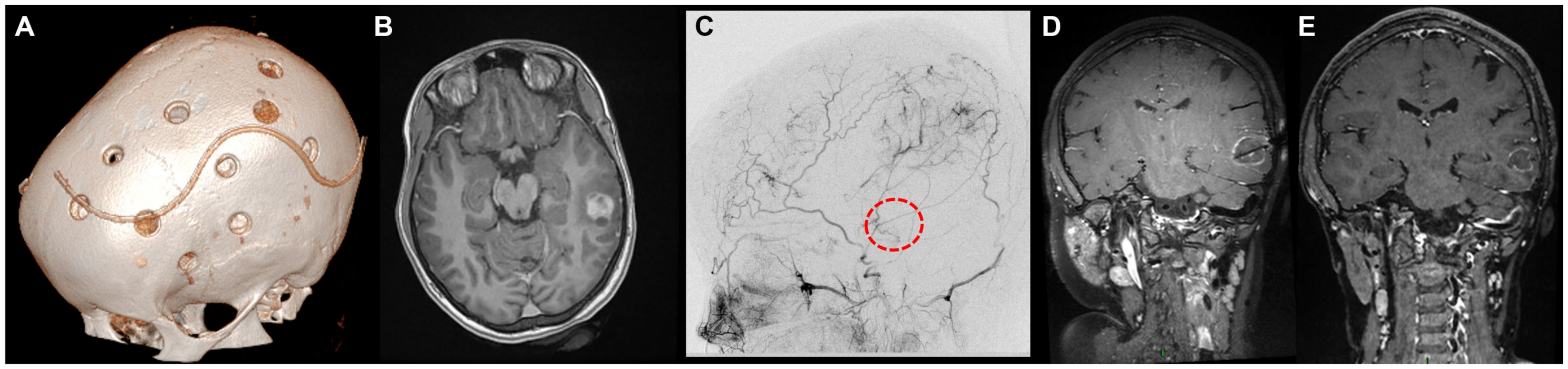
Panel showcasing the most relevant events in the medical history of L.M. **(A)** Day 1 postoperative cranial CT scan showing the position of the burr holes at the left side after revascularization surgery (9 years old). **(B)** Axial enhanced T1-weighted cerebral MRI at the age of 11, showing the appearance of a left middle temporal gyrus lesion, nodular with a small posterior cyst, surrounded by brain edema. **(C)** DSA of the branches of the external carotid artery showing the revascularization of the left hemisphere by the middle meningeal artery and posterior auricular artery. The red circle represents the putative location of the tumor. **(D)** Coronal enhanced T1-weighted cerebral MRI showing the trajectory of the LITT fiber at the end of the procedure. **(E)** Coronal enhanced T1-weighted cerebral MRI 10 months after the surgery, documenting the reduction in tumor size.

The first case had been diagnosed with familial NF1 associated to MMS and OPG. Following two strokes and recurrent transient ischemic attacks (TIAs), at the age of 9 years she underwent bilateral indirect revascularization through multiple burr hole (MBH) surgery (Figure 1A), conducted in children as described by our team (10, 11). In the postoperative course, no further stroke occurred, and 1-year postoperative digital subtraction angiography (DSA) showed Matsushima grade A revascularization. Two years later, follow-up MRI showed a left posterior middle temporal gyrus tumor suggestive of low-grade glioma, nodular with a small cystic component, homogeneously enhancing, that progressively increased in volume over the next 10 months (Figure 1B). After multidisciplinary discussion, we established surgical indication for tumor removal, given the tumor progression and the potential increased perioperative risks in case of further evolution. DSA showed collaterals from the middle meningeal and posterior auricular arteries supplying the left hemisphere (Figure 1C), and notably the supramarginal gyrus. These branches were above the theoretical location of the craniotomy for tumor resection. To avoid perioperative brain infarction and subsequent aphasia with open surgery, we performed an MR-guided (3 T General Electrics), stereotactic, robot-assisted (ROSA One Brain, Zimmer Biomet) laser ablation surgery (Visualase, Medtronic, United States) with a temporal trajectory, at the same time as a stereotactic biopsy (Figure 1D). Pathologic exam revealed a pilocytic astrocytoma. There were no perioperative complications, nor was there tumor recurrence after 10 months of follow-up (Figure 1E).

### Case 2

**Figure 2:**
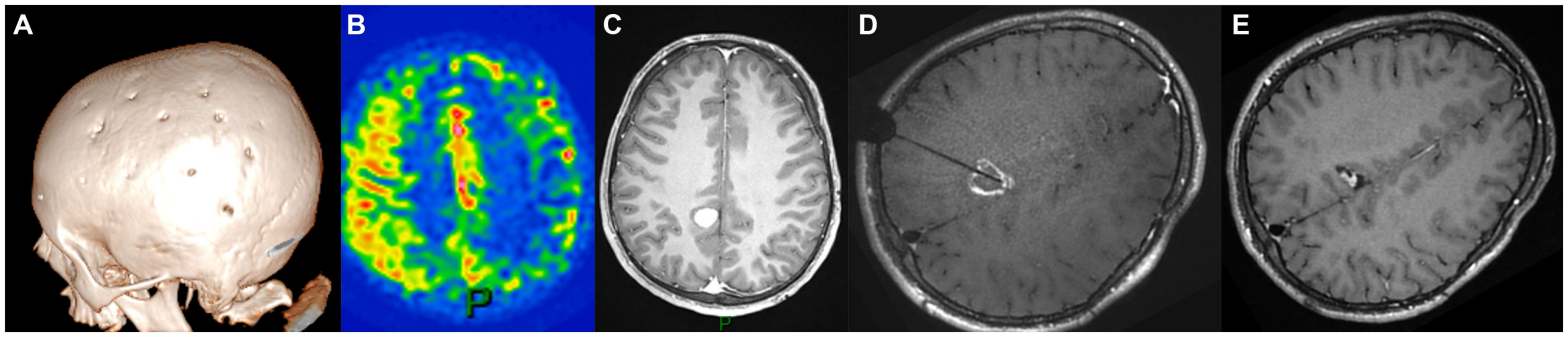
Panel illustrating the different steps of the treatment of E.B.S. **(A)** day-30 postoperative cranial CT scan showing the burr holes at the right side after revascularization surgery (6 years-old); **(B)** ASL-MRI of the brain showing cortical hyperperfusion at the level of frontoparietal burr holes and on the medial part of the hemispheres; **(C)** Axial enhanced T1-weighted cerebral MRI at the age of 15 documenting the appearance of a posterior cingular lesion, nodular and homogeneously enhancing; **(D)** Axial enhanced T1-weighted cerebral MRI showing the trajectory of the LITT fiber at the end of the procedure; **(E)** Axial enhanced T1-weighted cerebral MRI 12 months after the surgery displaying the reduction in tumor size.

The second case was that of a 6-year-old boy with familial NF1, who was diagnosed with unilateral severe MMD following focal seizures. He underwent left MBH surgery (Figure 2A) through a left coronal skin incision exceeding the midline, with no postoperative stroke and satisfactory revascularization of the left hemisphere on serial postoperative perfusion imaging (Figure 2B) with ASL MRI (12). Nine years later, follow-up MRI showed a right posterior cingular tumor suggestive of a low-grade glioma, nodular, homogeneously enhancing, and growing after 6 months (Figure 2C). As in the previously described case, the indication for tumor removal was discussed due to tumor progression and major perioperative risks in case of further volumetric increase. Open surgery was not deemed a safe option, as a biparietal skin incision and interhemispheric approach would have carried the risk of transosseous and transdural anastomosis damage over the right parietal lobe.

Therefore, we carried out MR-guided, stereotactic, robot-assisted laser ablation surgery (Visualase, Medtronic, United States) with a right intraparietal trajectory (Figure 2D). We did not perform a needle biopsy at the same time because of radiological features typical of low-grade glioma on MRI in the context of molecular diagnosis of NF1. No perioperative complications occurred, and the tumor size showed a significant reduction of 65% at 12-month follow-up (Figure 2E).

## Discussion

These two case reports demonstrate that LITT represents a good option for the ablation of supratentorial brain tumors in the context of NF1 after revascularization surgery with multiple burr holes. To our knowledge, this treatment strategy has not been described previously.

The association of moyamoya angiopathy and brain tumors outside the optic pathways is rare: only 4 NF1 patients (6–9) and 10 other cases (7 adults, 3 children) with concomitant CNS tumor and MMD have been described in the literature (13–19). As shown in Table 1, revascularization was carried out in only four of these patients (6, 9, 14, 19), mainly after brain tumor surgery. In the other reports, the vasculopathy occurred after tumor treatment (surgery or radiotherapy) or was not symptomatic at the time of tumor diagnosis. The 14-year-old NF1 patient reported by Arita et al. (6) is the sole patient who has undergone bilateral encephaloduroarteriosynangiosis (EDAS) 19 months before surgical biopsy of a thalamic malignant tumor. In this case, the authors used a small craniotomy with a neuronavigation-guided needle biopsy to avoid damage to dural collaterals.

**Table 1:** Review of the literature on the co-occurrence of moyamoya angiopathy and CNS brain tumor.

Author and year	NF1	Age (years)	Tumor location	Histology	Revascularization *(timing)*	Surgical strategy	Complications	Comments
Mori et al., 1978	No	12	Sellar region	Craniopharyngioma	None	Debulking	Perioperative death	MM onset after RT
		34	Sellar region	Germinoma	None	Debulking	Perioperative death	MM onset after RT
		3	Basal ganglia	PA	None	Debulking	None	MM onset after RT
		9	Sellar region	Craniopharyngioma	None	Debulking	None	
Lau et al., 1986	No	3	Sellar region	Craniopharyngioma	None	Debulking	None	
Aihara et al., 1992	No	4	Cerebellar vermis	PA	None	Debulking	None	Syndromic features
Arita et al., 1992	No	40	Sellar region	Adenoma	None	STR	None	Surgery refused
		23	Sellar region	N.A.	None	None		
Kitano et al., 2000	No	8	Brainstem	PA	Bilateral double STA–MCA bypass + EMS *(after tumor surgery)*	PR	None	
Shibata et al., 2010	No	15	Sellar region	Germinoma	None	Open biopsy	None	Good response to CT + RT
Horiguchi et al., 2011	Yes	32	Thalamus	PXA	None	Open biopsy + VPS	Meningitis due to shunt infection	Died after 18 months
Arita et al., 2013	Yes	14	Thalamus	GBM	Bilateral EDAS *(before tumor surgery)*	Craniotomy + needle biopsy	None	Died after 30 months
Gold et al., 2013	Yes	17	Cervical spinal cord	PA	EDMS *(simultaneous with tumor surgery)*	STR	None	
Xu et al., 2018	No	46	Tuberculum sellae	Meningioma	EDMS *(simultaneous with tumor surgery)*	GTR	None	
Tanioka et al., 2022	Yes	48	Frontal lobe	GBM	None	GTR	Neurogenic pulmonary edema	

CT, chemotherapy; EDAS, encephaloduroarteriosynangiosis; EDMS, encephaloduromyosynangiosis; GBM, glioblastoma multiforme; GTR, gross total resection; MCA, middle cerebral artery; PA, pilocytic astrocytoma; PR, partial resection; PXA, pleomorphic xanthoastrocytoma; RT, radiotherapy; STA, superficial temporal artery.

Treating a brain tumor in the context of NF1 and moyamoya is complicated and bears a high risk of stroke, because on the one hand, radiotherapy exacerbates the vasculopathy (20, 21), and on the other hand, microsurgery may require interrupting spontaneous transdural collaterals; medical treatments with chemotherapy or MEK inhibitors may be an active alternative, but the long duration of treatment, the burden of side effects, and the risk of tumor recurrence after treatment discontinuation lead us to prefer a radical local treatment whenever possible.

At our center, indirect revascularization is performed via the multiple burr hole technique, because bilateral revascularization can be carried out in a single procedure, and because burr holes are realized all over the cranial vault, offering whole hemispheric revascularization, and ultimately excellent (>90%) stroke control (10, 12). However, brain collaterals will inevitably occur in the vicinity of any surgical access if a craniotomy is further needed.

LITT can therefore represent the first choice procedure in these cases, given that: (i) it is performed in stereotactic conditions through a 3.2 mm burr hole, with an entry point that can be chosen according to preoperative MRI and DSA to avoid any vessel; (ii) a stereotactic needle biopsy can be performed during the same procedure, providing further treatment possibilities according to the molecular profile if required; (iii) it is performed under real-time MR imaging, allowing simulation of the ablation volume and tailoring of the therapy; (iv) it can be repeated along the same or another trajectory in case of relapse. Provided that the lesion conformation is favorable (a round or elliptic shape), LITT may achieve satisfactory ablation volumes (22) and assure sustained control of the tumor (4, 5): this is particularly attractive in NF1 patients to reduce the need for radiation and prolonged medical treatment, recognizing that the use of radiotherapy is not recommended (20, 21, 23). The choice of whether or not to perform a stereotactic needle biopsy before the introduction of the laser fiber is still under discussion, as it has been pointed out that this could cause MRI artifacts at the biopsy site, resulting in sub-optimal MR thermometry and potentially decreased LITT efficacy (24, 25). A recent retrospective registry-based comparative study involving 678 patients with malignant brain tumors undergoing LITT alone and 373 cases treated through needle biopsy before LITT did not indicate differences in the safety profile (26).

Finally, tumoral size may limit LITT indications, both because the whole tumor may not be ablated, and because the risk of complications is correlated to the tumor size and has been found to reach 26.7% in a multicentric series, with a 5.8% risk of neurological deficits and 2.3% risk of mortality (5). This might justify prolonged MRI follow-up of NF1 patients with previously revascularized MMS, with the aim of detecting tumors when they are morphologically and volumetrically suitable for laser ablation.

## Conclusion

Association of moyamoya angiopathy and brain neoplasms is rare but may occur, notably in neurofibromatosis type 1. Avoiding stroke while preserving EC–IC anastomosis is a challenge that LITT can overcome, provided that the tumor location and volume are suitable. This increases the potential indications for this technique, proposed here as an alternative to irradiation (not recommended in the context of NF1 and moyamoya) or prolonged medical treatment.

## Data availability statement

The original contributions presented in the study are included in the article/supplementary material, further inquiries can be directed to the corresponding author.

## Ethics statement

The institutional review board (Assistance Publique Hôpitaux de Paris) authorized the waiver of written informed consent for this study because of its non-interventional retrospective design, and patients were informed they could oppose the use of their health-related data for research purposes. The studies were conducted in accordance with the local legislation and institutional requirements. Written informed consent was obtained from the minor(s)’ legal guardian/next of kin for the publication of any potentially identifiable images or data included in this article.

## Author contributions

LG: Conceptualization, Formal analysis, Investigation, Methodology, Visualization, Writing – original draft, Writing – review & editing. KB: Writing – review & editing. SB: Writing – review & editing. MK: Writing – review & editing. ON: Writing – review & editing. MB: Writing – review & editing. FB: Writing – review & editing. SA: Writing – review & editing. VD-R: Writing – review & editing. NB: Writing – review & editing. TB: Funding acquisition, Supervision, Validation, Writing – review & editing.
